# UPA-seq: prediction of functional lncRNAs using differential sensitivity to UV crosslinking

**DOI:** 10.1261/rna.067611.118

**Published:** 2018-12

**Authors:** Taiwa Komatsu, Saori Yokoi, Koichi Fujii, Mari Mito, Yusuke Kimura, Shintaro Iwasaki, Shinichi Nakagawa

**Affiliations:** 1 RNA Biology Laboratory, Faculty of Pharmaceutical Sciences, Hokkaido University, Sapporo 060-0812, Japan; 2 Graduate School of Science and Engineering, Saitama University, Saitama 338-8570, Japan; 3 RNA Systems Biochemistry Laboratory, RIKEN Cluster for Pioneering Research, Saitama 351-0198, Japan; 4 Department of Computational Biology and Medical Sciences, Graduate School of Frontier Sciences, The University of Tokyo, Chiba 277-8561, Japan

**Keywords:** noncoding RNAs, lncRNAs, RNA-binding proteins, UV crosslinking, eCLIP, RNA-seq, phenol-chloroform extraction

## Abstract

While a large number of long noncoding RNAs (lncRNAs) are transcribed from the genome of higher eukaryotes, systematic prediction of their functionality has been challenging due to the lack of conserved sequence motifs or structures. Assuming that some lncRNAs function as large ribonucleoprotein complexes and thus are easily crosslinked to proteins upon UV irradiation, we performed RNA-seq analyses of RNAs recovered from the aqueous phase after UV irradiation and phenol-chloroform extraction (UPA-seq). As expected, the numbers of UPA-seq reads mapped to known functional lncRNAs were remarkably reduced upon UV irradiation. Comparison with ENCODE eCLIP data revealed that lncRNAs that exhibited greater decreases upon UV irradiation preferentially associated with proteins containing prion-like domains (PrLDs). Fluorescent in situ hybridization (FISH) analyses revealed the nuclear localization of novel functional lncRNA candidates, including one that accumulated at the site of transcription. We propose that UPA-seq provides a useful tool for the selection of lncRNA candidates to be analyzed in depth in subsequent functional studies.

## INTRODUCTION

A large number of long noncoding RNAs (lncRNAs) are transcribed from the genomes of higher eukaryotes, and accumulating evidence suggests that lncRNAs are involved in a variety of molecular processes, including the epigenetic regulation of gene expression, formation of nonmembranous cellular bodies, and sequestration of miRNAs or RNA-binding proteins (RBPs) (for reviews, see Quinn and Chang 2016; Wu et al. 2017; Kopp and Mendell 2018). While the number of lncRNA genes (15,877) is almost comparable to the number of protein-coding genes (19,881) according to the most recent statistics of human gene annotation (GENCODE GRCh38, http://www.gencodegenes.org/stats.html), their functional annotations are far from complete. For a vast majority of lncRNAs identified to date, our knowledge is limited to their expression patterns and primary sequences (for review, see de Hoon et al. 2015). Considering that certain fractions of lncRNAs might be “transcriptional noise” produced via stochastic associations of RNA polymerase with open chromatin regions (for review, see Struhl 2007), it is necessary to distinguish physiologically relevant lncRNAs from “junk” RNAs. While recent studies identified nuclear-localizing elements in lncRNAs (Zhang et al. 2014; Lubelsky and Ulitsky 2018), lncRNAs commonly lack conserved sequence motifs or secondary structures, making functional classification of novel lncRNAs rather challenging. This situation is largely different from the case of proteins, which can be systematically categorized into families of molecules according to their individual domain structures (for review, see Hirose and Nakagawa 2016).

Recently, a genome-wide CRISPR-interference screen identified hundreds of lncRNAs that affect cellular growth (Liu et al. 2017). Another more specific, genome-scale CRISPR–Cas9 activation screening identified 11 lncRNAs that confer drug resistance in tumor cells (Joung et al. 2017). Further functional lncRNAs might be identified when we can establish appropriate cell-based scalable assays that correctly evaluate the biological functions of lncRNAs in cultured cell lines. On the other hand, lncRNAs tend to display more tissue- and cell-type-specific expression patterns (Cabili et al. 2011; Derrien et al. 2012), and their cellular and physiological functions can possibly be addressed only under particular conditions and in certain contexts, some of which might be hard to replicate using commonly available cultured cell lines. In addition, the phenotypes of certain lncRNAs obtained by cellular studies do not always correlate with the phenotypes observed in mutant animals (for review, see Nakagawa 2016). Thus, it is essential to perform detailed physiological analyses focusing on specific lncRNAs using mutant animals to understand the biological roles of lncRNAs (for reviews, see Trapnell et al. 2013; Bassett et al. 2014). Although recent advancements in genome-editing technologies have enabled rapid generation of genetically modified animals and accelerated reverse-genetic approaches (for review, see Burgio 2018), the number of lncRNAs expressed from the genome far exceeds the number of genes that can be feasibly studied using mutant animals. Accordingly, it is important to develop a method that can efficiently identify candidate lncRNAs for subsequent extensive physiological analyses.

Most of the functional lncRNAs examined to date exert their functions through direct association with multiple proteins. Xist, a key factor that controls X chromosome inactivation, associates with a number of RBPs, including hnRNP U, hnRNP K, and Rbm15, as well as transcriptional regulators such as Spen (Hasegawa et al. 2010; Chu et al. 2015; McHugh et al. 2015; Monfort et al. 2015). A number of lncRNAs have also been demonstrated to associate with chromatin-modifying complexes to regulate epigenetic gene expression (for review, see Holoch and Moazed 2015). Neat1 functions as an architectural component of nuclear bodies called paraspeckles (Chen and Carmichael 2009; Clemson et al. 2009; Sasaki et al. 2009; Sunwoo et al. 2009) and builds a characteristic core-shell structure through association with DBHS family members of RBPs and a spectrum of RBPs with prion-like domains (PrLDs), including Fus, Tardbp, and Rbm14 (Fox et al. 2002; Souquere et al. 2010; Naganuma and Hirose 2013; Hennig et al. 2015; West et al. 2016). These two nuclear lncRNAs, as well as Malat1 and Gomafu/MIAT, which associate with splicing factors (Tano et al. 2010; Tripathi et al. 2010; Tsuiji et al. 2011) form extremely large RNA-protein complexes and are sedimented into fractions heavier than polysomes by sucrose density gradient ultracentrifugation (Ishizuka et al. 2014). NORAD, a cytoplasmic lncRNA that controls genome stability, also associates with an RBP, Pumilio through tandem arrays of its binding motif (Lee et al. 2016; Tichon et al. 2016). These observations led us to hypothesize that the association of multiple proteins might be regarded as a hallmark of functional lncRNAs that exert their functions as ribonucleoprotein complexes.

Some time ago, a simple method termed FAIRE-seq was developed and enabled the identification of nucleosome-free regions of the genome associated with regulatory proteins (Giresi et al. 2007). FAIRE-seq utilizes the differential biochemical properties of nucleic acid and proteins during a phenol-chloroform extraction: DNA fragments crosslinked to proteins are separated into the interphase that contains denatured proteins, whereas free DNA is separated into the aqueous phase and is efficiently recovered by ethanol precipitation for subsequent analysis by deep sequencing (Giresi et al. 2007). The success of this method encouraged us to take a similar approach to develop a method that enables the prediction of the functionality of lncRNAs based on differential sensitivity to UV irradiation, which induces covalent bonds between nucleic acids and closely associated proteins (Greenberg 1979; Wagenmakers et al. 1980). In this study, we irradiated cells with UV and performed RNA-seq analyses using RNAs purified by conventional acid guanidinium phenol-chloroform extraction (UPA-seq: UV-phenol aqueous phase RNA sequencing). We found that known functional lncRNAs including Xist, Neat1, Malat1, and Gomafu/Miat were efficiently crosslinked to proteins and were largely depleted from the aqueous phase upon UV irradiation, leading to a dramatic decrease in the number of mapped reads obtained by UPA-seq. On the other hand, the number of reads mapped to Blustr and Upperhand, for which transcription but not transcribed products are important for function (Anderson et al. 2016; Engreitz et al. 2016), were not affected by UV irradiation. We also examined the subcellular distribution of novel functional lncRNA candidates identified by the decrease in UPA-seq reads and found that some of them formed a characteristic localization at their putative transcription sites. We propose UPA-seq as a useful tool to select candidate lncRNAs prior to intensive functional analyses.

## RESULTS

### Recovery of known representative functional lncRNAs from the aqueous phase is greatly reduced upon UV irradiation

**FIGURE 1.**
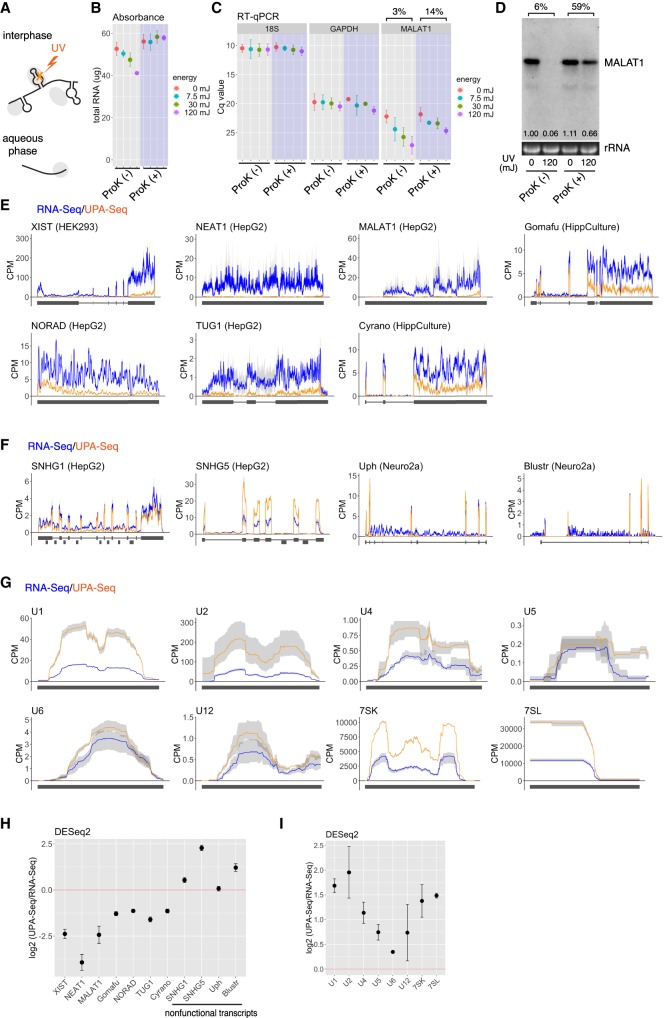
Reduced recovery of known functional lncRNAs from the aqueous phase upon UV irradiation. (*A*) Schematic drawings of the principle of UPA-seq. UV irradiation introduces crosslinking between RNA (black lines) and proteins (gray ovals), and crosslinked complexes are separated into the interphase after phenol-chloroform extraction. (*B*) The amount of total RNA (µg) recovered from the aqueous phase upon different doses of UV irradiation (0, 7.5, 30, and 120 mJ/cm^2^) with or without pretreatment with proteinase K [ProK(+)/ProK(−)]. Data are presented as the means ± SD of three biological triplicates. 18S: 18S ribosomal RNA. (*C*) RT-qPCR quantification of RNA transcripts recovered from the aqueous phase upon different doses of UV irradiation (0, 7.5, 30, and 120 mJ/cm^2^) with or without pretreatment with proteinase K [ProK(+)/ProK(−)]. The Cq values of the representative functional lncRNA MALAT1 are increased upon UV irradiation, which was partially restored by the proteinase K pretreatment. Data are presented as the mean ± SD of five biological replicates except for the 7.5 mJ condition, which is presented as biological duplicates. Note that the scale Y is reversed. Fold change upon UV irradiation at 120 mJ (3%, 14%) was calculated by 2^−ΔCq^. (*D*) Northern blot analyses of MALAT1 recovered from the aqueous phase upon UV irradiation (120 mJ/cm^2^) with or without pretreatment with proteinase K [ProK(+)/ ProK(−)]. The numbers in the blot area indicate the relative expression quantified by ImageJ. (*E*) Distribution of UPA-seq and RNA-seq reads mapped to representative functional lncRNAs. Blue and orange lines represent the distribution of reads obtained by UPA-seq and RNA-seq, respectively. Cell types used for the analysis are indicated by the name of the lncRNA genes. (*F*) Distribution of UPA-seq and RNA-seq reads mapped to lncRNAs, of which transcribed products are thought to be nonfunctional. (*G*) Distribution of UPA-seq and RNA-seq reads mapped to classic ncRNAs. (*H*) Log_2_ fold change of reads obtained by UPA-seq and conventional RNA-seq mapped to lncRNAs. Sequence reads were counted by featureCount and normalized by DESeq2. Note that nonfunctional lncRNA transcripts do not exhibit decreased recovery. (*I*) Log_2_ fold change of reads mapped to classic ncRNAs upon UV irradiation. Data are presented as the means ± SD of three biological triplicates. CPM in E-G represents counts per millions of reads.

To investigate whether lncRNAs tightly associate with multiple RBPs, we focused on UV irradiation, which induces the formation of covalent bonds between RNAs and RBPs at low efficiency (Greenberg 1979; Wagenmakers et al. 1980). Because of the low efficiency of UV crosslinking, we speculated that lncRNAs that weakly associate with interacting proteins remain uncrosslinked under mild UV irradiation conditions. However, functional lncRNAs that form tight ribonucleoprotein complexes are expected to be crosslinked to at least one of the associating proteins, which are then co-fractionated into the interphase after phenol-chloroform extraction (Fig. 1A). To test these ideas, we first irradiated HepG2 cells with UV_260_ (7.5, 30, and 120 mJ/cm^2^) and examined the amount of RNA recovered from the aqueous phase after conventional acid guanidinium thiocyanate-phenol-chloroform (AGTPC) extraction. The total amount of RNA obtained from a fixed number of UV-irradiated (120 mJ/cm^2^) and nonirradiated HepG2 cells was 52.6 ± 3.2 µg and 41.1 ± 0.7 µg, respectively (Fig. 1B), suggesting that 22% of total RNA was crosslinked to certain proteins and failed to be recovered from the aqueous phase. The reduced recovery was restored by proteinase K pretreatment before AGTPC extraction (Fig. 1B), further confirming that protein-crosslinked RNAs were fractionated into the interface. Subsequent RT-qPCR analyses revealed that functional lncRNA such as MALAT1 was reduced upon UV irradiation in a dose dependent manner, whereas the mRNA of a housekeeping gene GAPDH was much less affected (Fig. 1C). Interestingly, 18S ribosomal RNAs were also less affected than known functional lncRNAs (Fig. 1C), which was consistent with a classic study showing that ribosomal RNAs are less efficiently crosslinked to proteins than other heterologous RNAs (Wagenmakers et al. 1980). The reduction of MALAT1 upon UV irradiation was partially restored by pretreatment with proteinase K (Fig. 1C), the effects of which were more prominent when these transcripts were detected on the northern blot; fold change upon UV irradiation in proteinase K-treated samples were 14% by qPCR and 59% by northern blot, respectively (Fig. 1C,D). These observations suggest that covalent bond formation introduced by UV irradiation interfered with the reverse transcription reaction, resulting in greater reduction upon UV irradiation when detected by qPCR. UV irradiation did not induce the fragmentation of RNAs, as confirmed by the gel electrophoresis pattern of rRNA or MALAT1 (Fig. 1D).

To further investigate the sensitivity of lncRNAs to UV irradiation systematically, we performed deep sequencing analysis of the RNAs recovered from the aqueous phase after UV irradiation (UPA-seq) using multiple cultured cell, including Neuro2a, the human hepatocellular carcinoma cell line HepG2, and the human embryonic kidney cell line HEK293, as well as primary cultures of mouse hippocampal neurons (HippCulture). As a control, we performed conventional RNA sequencing (RNA-seq) using nonirradiated cells. Comparison of the sequence reads obtained by RNA-seq and UPA-seq revealed that the number of sequence reads mapped to representative functional lncRNAs, including Xist, Neat1, Malat1, Gomafu, NORAD, TUG1, and Cyrano, were greatly reduced after UV irradiation (Fig. 1E,H). On the other hand, the UPA-seq reads that mapped to nonfunctional lncRNAs, such as host genes for snoRNA, SNHG1 and SNHG4, were unchanged or rather increased (Fig. 1F,H). In addition, UPA-seq reads that mapped to the exonic regions of Blustr and Upperhand (Uph) were also unchanged (Fig. 1F,H), consistent with a previous proposal that the transcription of these lncRNAs, but not their transcribed RNA products, are essential for their biological functions (Anderson et al. 2016; Engreitz et al. 2016). We also examined the fold change of reads that mapped to classic noncoding RNAs associated with the housekeeping process of gene expression, including UsnRNAs, 7SK, and 7SL (Fig. 1G,I). Unexpectedly, the sequence reads obtained by UPA-seq were unchanged or even increased compared to those obtained by conventional RNA-seq in all of the cases (Fig. 1G,I), suggesting that these “functional” noncoding RNAs are less likely to be UV-crosslinked to associating proteins under the irradiation condition, as was the case for ribosomal RNAs previously reported (Wagenmakers et al. 1980).

### Fewer sequence reads are mapped to lncRNAs in UPA-seq

**FIGURE 2.**
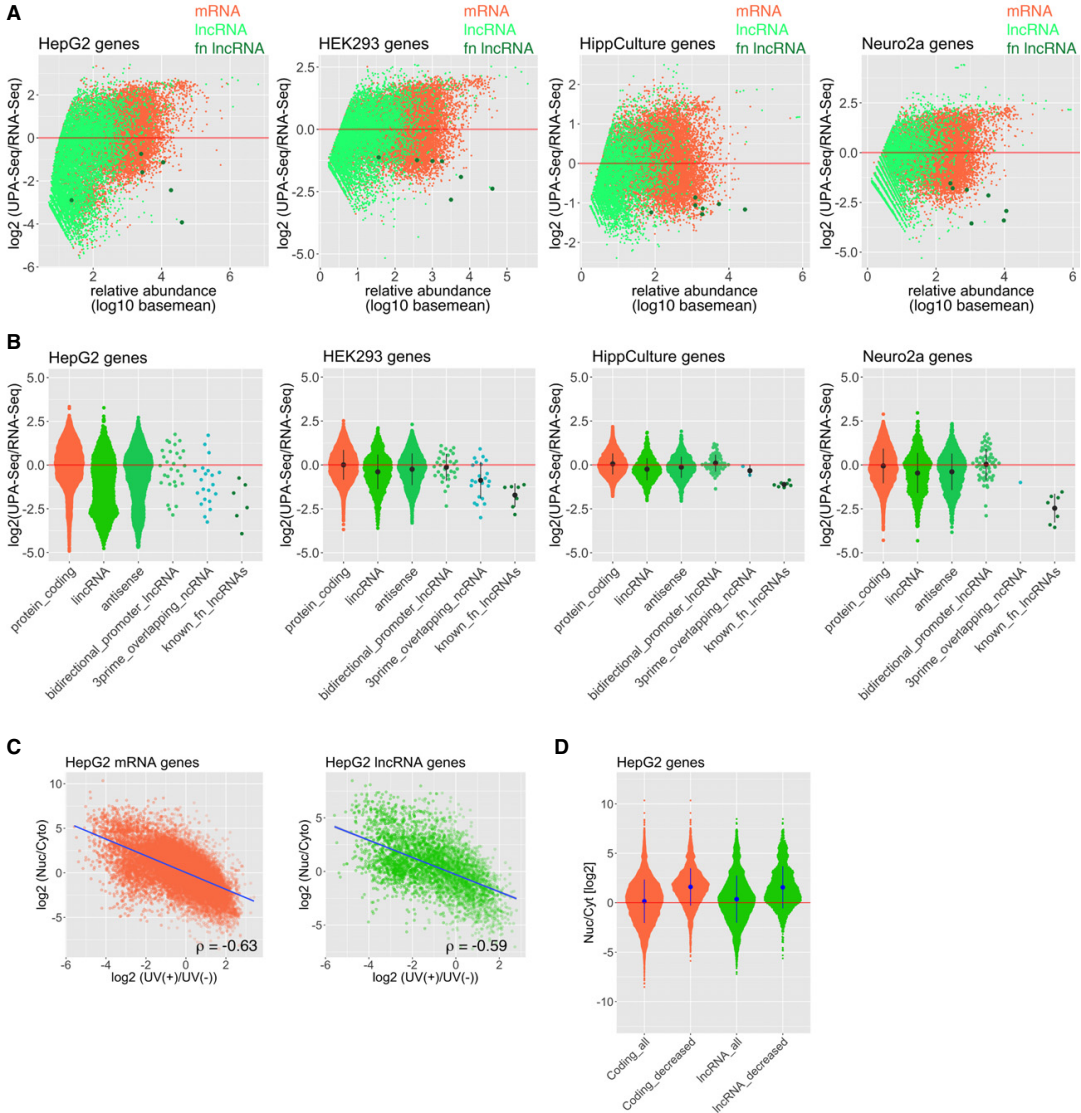
Decreased RNA-seq reads mapped to known functional lncRNAs upon UV irradiation. (*A*) MA-plot of log_2_ fold change of reads upon UV irradiation as a function of the relative abundance (log_10_ baseMeans) calculated by DESeq2 in HepG2, HEK293, Neuro2a, and HippCulture cells. Light green dots represent lnRNAs, coral dots represent mRNAs, and dark green dots represent known functional lncRNAs (Xist, Neat1, Malat1, Mia, NORAD, TUG1, and Cyrano). Some of the functional lncRNAs that are not expressed in each cell type are not shown in the panels. fn lncRNA: functional lncRNA. (*B*) Quasi-random beeswarm plots of log_2_ fold change of reads upon UV irradiation in HepG2, HEK293, Neuro2a, and HippCulture cells. Bar plots indicate the means ± SD of three biological triplicates. Note that lncRNAs generally exhibit negative fold change. known_fn_lncRNAs: known functional lncRNAs. (*C*) Scatter plots illustrating the relationship between the fold change of reads upon UV irradiation and the ratio of reads in the nuclear/cytoplasmic fractions in HepG2 cells. The correlation coefficient (ρ) was calculated by the Spearman's rank correlation analyses. (*D*) Quasi-random beeswarm plots of the ratio of reads in the nuclear/cytoplasmic fractions of HepG2 cells. Bar plots indicate the means ± SD of three biological triplicates. Note that the mRNAs/lncRNAs that exhibited a decrease in UPA-seq reads are preferentially enriched in the nuclear fraction.

We then examined the genome-wide fold change of reads mapped to lncRNAs and mRNAs upon UV irradiation (Fig. 2A). For this analysis, lncRNAs were defined when they had the GENCODE gene/transcript type annotations “lincRNA,” “antisense,” “bidirectional_promoter_lncRNA,” or “3prime_overlapping_ncRNA.” As expected, the number of UPA-seq reads mapped to representative functional lncRNAs (Xist, Neat1, Malat1, Miat, NORAD, TUG1) was remarkably decreased in all of the four cell types we examined (dark green dots in Fig. 2A), providing a rationale to select novel functional lncRNA candidates by the fold change of reads after UV irradiation. We also examined the overall fold change of reads mapped to different GENCODE gene categories upon UV irradiation (Fig. 2B). The number of UPA-seq reads mapped to lncRNAs annotated as “lincRNA” or “antisense” exhibited a significant decrease relative to the counts mapped to protein-coding mRNAs, suggesting that lncRNAs are generally associated with more protein than mRNAs. Notably, the fold change of reads mapping to lncRNAs in HepG2 cells exhibited a bimodal distribution with two peaks, one at approximately zero and the other at approximately −2.6 (Fig. 2B), which might represent a group of nonfunctional and functional lncRNAs, respectively. The bimodal distribution of the fold change was also recognizable in Neuro2a cells but not in HEK293 or HippCulture cells, possibly reflecting a differential sensitivity to UV irradiation in each cell type.

We then examined if nuclear RNAs show a greater decrease in UPA-seq reads compared to cytoplasmic RNAs. The ratio of nuclear/cytoplasmic RNA (Nuc/Cyto ratio) for each transcript in HepG2 cells was calculated using the ENCODE RNA-seq data set and was compared with the fold change of reads upon UV irradiation (Fig. 2C). We observed a moderate correlation between the two values both for lncRNAs and mRNAs (Spearman's rank correlation coefficient ρ = 0.59 for lncRNAs and ρ = 0.63 for mRNAs, Fig. 2C), suggesting that nuclear RNAs associate with more proteins compared to cytoplasmic RNAs. We also compared the average Nuc/Cyto ratio of all gene transcripts and a group of gene transcripts that exhibited a decrease (log_2_ fold change < 0, FDR <0.01) in UPA-seq reads (Fig. 2D). We confirmed the statistically significant (*P* < 2.2 × 10^−16^) increase in the Nuc/Cyto ratio of both the mRNAs and lncRNAs with decreased UPA-seq reads (Fig. 2D), further supporting that nuclear RNAs associate with more proteins compared to cytoplasmic populations.

### Weak correlations between the length or expression levels of lncRNAs and the decrease in UPA-seq read counts

**FIGURE 3.**
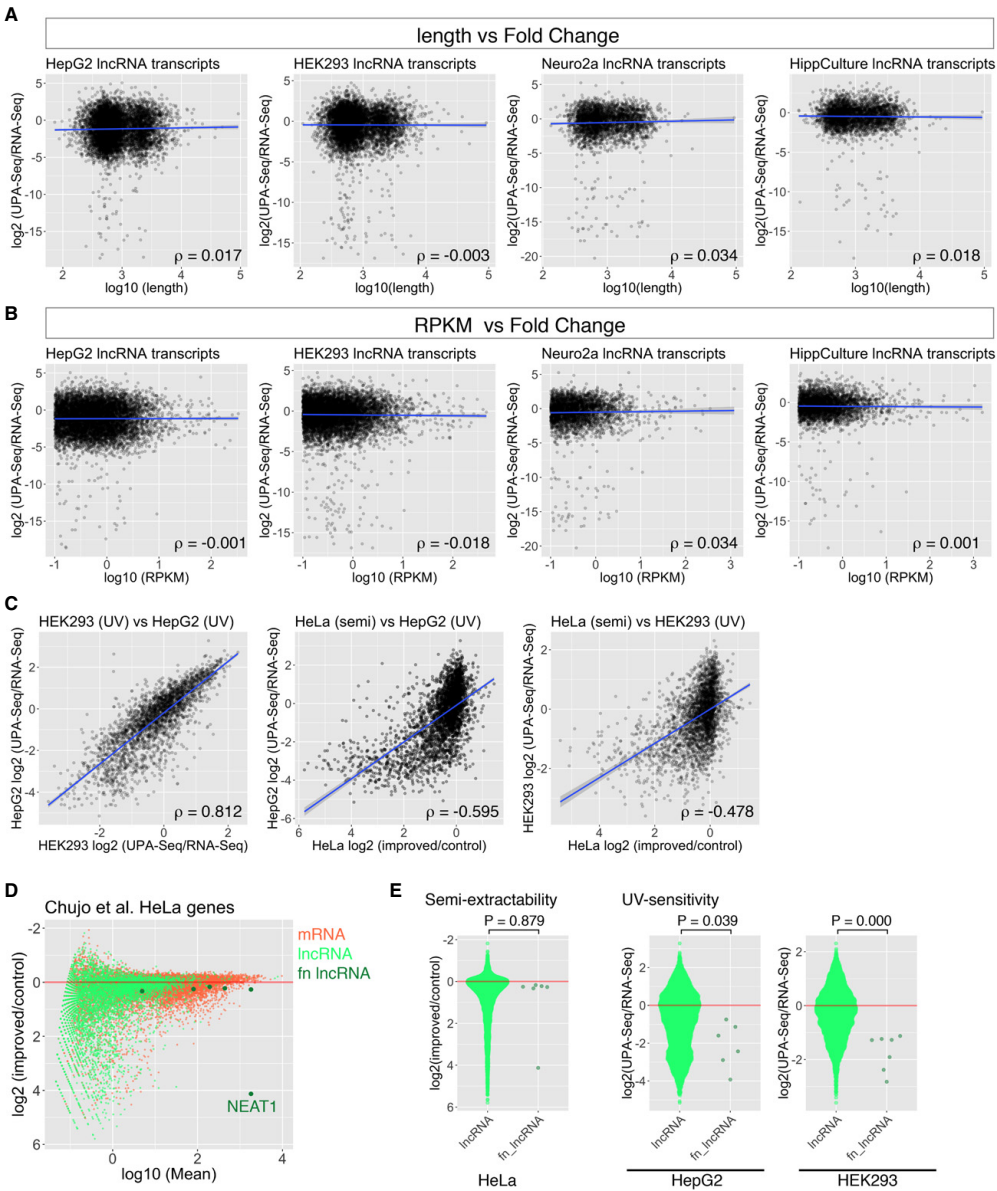
Correlation analysis of fold change of reads upon UV irradiation and the length, RPKM, and semi-extractive properties of lncRNAs. (*A*,*B*) Scatter plots illustrating the relationship between the length (*A*) or RPKM (*B*) of lncRNAs and the fold change of reads upon UV irradiation in HepG2, HEK293, Neuro2a, and HippCulture cells. Correlation coefficient (ρ) was calculated by Spearman's rank correlation analyses. Note that the length or RPKM did not exhibit a correlation with the fold change of reads upon UV irradiation. Blue lines represent regression lines. (*C*) Scatter plots illustrating the relationship between the fold change of reads in two different cell types (HEK293 [UV], HepG2 [UV]) and the fold change of reads after improved extraction (HeLa [semi]) compared with the fold change of reads upon UV irradiation (HepG2 [UV], HEK293 [UV]). Note that the *y*-axes HeLa (semi) showing the fold change of reads after improved extraction are reversed. Correlation coefficient (ρ) was calculated by Spearman's rank correlation analyses. Blue lines represent regression lines. (*D*) MA-plot of log_2_ fold change of reads upon improved extraction as a function of the relative abundance (log_10_ baseMeans) calculated by DESeq2 with an inverted *y*-axis. Light green dots represent lnRNAs, coral dots represent mRNAs, and dark green dots represent known functional lncRNA genes (fn lncRNA). Note that the extraction of NEAT1 is dramatically enhanced by the improved extraction, whereas other representative functional lncRNAs exhibited only mild, if any, enhancement. (*E*) Quasi-random beeswarm plots of log_2_ fold change of reads upon improved extraction (semi-extractability) and UV irradiation (UV sensitivity). Cell types used are indicated at the *bottom* of the plot. *P*-values are calculated by Wilcoxon rank sum test. Note that the *y*-axis for the semi-extractability in HeLa cell is reversed.

Because all of the representative functional lncRNAs mentioned above are relatively long and abundantly expressed, the decrease in UPA-seq reads might be simply explained by increased stochastic interactions between proteins and long, abundant lncRNAs. To test whether this is the case or not, we compared the length and abundance of each lncRNA transcript with the fold change of reads obtained by UPA-seq and RNA-seq in HepG2 cells (Fig. 3A,B). The Spearman's rank correlation coefficient (ρ) between the length and the fold change of reads in HepG2 cells was −0.0017 (Fig. 3A), suggesting that the decrease in UPA-seq reads was independent of the length of lncRNAs. We also failed to detect a correlation between the fold change of reads and the expression level of lncRNAs estimated by reads per kilobase of transcript per million mapped reads (RPKM) (ρ = 0.001, Fig. 3B). The same trend was observed for all the cell types we used (HEK293, Neuro2a, and HippCulture) (Fig. 3A,B). These observations suggested that the sensitivity to UV crosslinking is controlled by the amount of RBPs that recognize specific sequences or the secondary structures of lncRNAs rather than by sequence-independent stochastic association with surrounding proteins.

Recently, Chujo et al. discovered that lncRNAs, including NEAT1, that form certain nuclear bodies are hard to purify by conventional AGTPC treatment, and they collectively called them as “semi-extractable” lncRNAs (Chujo et al. 2017). Improved extraction methods such as needle sharing or heat treatment dramatically increased the recovery of semi-extractable lncRNAs, and this semi-extractability has been proposed as a characteristic of architectural lncRNAs that function in nuclear bodies (Chujo et al. 2017). We were interested how these semi-extractable lncRNAs were represented in UPA-seq read counts, and we compared our results with the data set reported in the previous study (Chujo et al. 2017). Because the authors used the HeLa cell line, which was different from the cell lines we used, we initially compared the fold change of reads obtained from HepG2 cells and HEK293 cells in our study. Despite the difference in the cell line, we observed a strong correlation between the two values (Fig. 3C, left panel, ρ = 0.812 in Spearman's rank correlation analyses), suggesting that the fold change of reads mapped to certain lncRNAs upon UV irradiation is fairly conserved across different cell lines. We then compared the fold change of reads with an improved extraction method and the fold change of reads upon UV irradiation (Fig. 3C, middle and right panels). We observed a fairly good negative correlation between the two values (ρ = −0.595 in HepG2 cells and ρ = −0.4878 in HEK293 cells), suggesting that the decrease in the read counts in UPA-seq could also be used to predict semi-extractable, architectural lncRNAs. We note that this observation may even be underestimated because of the use of different cell lines for each approach. Notably, the recovery of many of the representative functional lncRNAs, including MALAT1, NORAD, CYRANO, TUG1, and MIAT, were not dramatically affected by the improved extraction, whereas they exhibited a remarkable reduction in UPA-seq (Fig. 3D,E). Accordingly, compared to previous methods, UPA-seq may be applicable for the prediction of a wider range of functional lncRNAs.

### Nuclear localization of functional lncRNA candidates identified by UPA-seq

**FIGURE 4.**
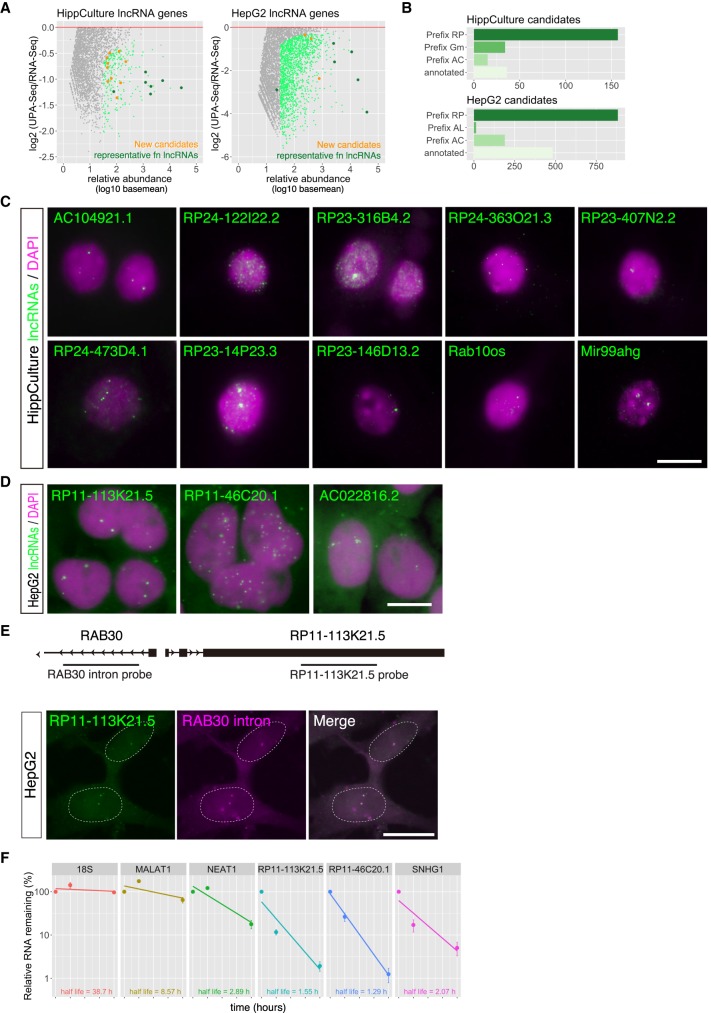
Identification of novel functional lncRNA candidates by UPA-seq. (*A*) MA-plot of log_2_ fold change of reads upon UV irradiation in HippCulture and HepG2 cells as a function of the relative abundance (log_10_ baseMeans). Light green dots represent lncRNAs that exhibited significant (FDR < 0.05) fold change, orange dots represent functional lncRNA candidates used for the following FISH studies, and dark green dots represent known functional lncRNAs. Note that only lncRNAs with negative log_2_ fold change values are illustrated. (*B*) Group of functional lncRNA candidates that exhibited significant decrease (FDR < 0.05) upon UV irradiation. Genes are categorized into known genes (annotated) and unknown genes assigned with ID numbers with various prefixes (RP, Gm, AC, and AL). (*C*,*D*) FISH images showing the subcellular distribution of functional lncRNA candidates (green) in HippCulture cells (*C*) and HepG2 cells (*D*). Magenta denotes nuclei counterstained with DAPI. Scale bar, 10 µm. (*E*) Schematic illustration showing the gene organization at the Rab30/RP11-113K21.5 locus and the positions of the probes used for simultaneous detection. Note that the signals obtained with probes that detect RP11-113K21.5 were closely associated with the signals obtained with probes that detect Rab30 intron sequences. Dashed lines represent the position of the nucleus. Scale bar, 10 µm. (*F*) Half-lives of 18S rRNA, MALAT1, NEAT1, RP11-113K21.5, RP11-46C20.1, and SNHG1, as measured by BRIC RT-qPCR. Note that the functional lncRNA candidates exhibited relatively short half-lives.

We then attempted to identify novel functional lncRNA candidates using UPA-seq, initially focusing on genes expressed in HippCulture cells. We selected 242 moderately abundant lncRNAs that exhibited decreased read counts in UPA-seq (log_10_ [baseMean] >1.5, FDR < 0.05) in HippCulture cells (Fig. 4A). The majority (86%) of these lncRNAs with GENCODE assigned gene names containing the prefix RP, Gm, or AC or the suffix Rik have not been described in the literature (Fig. 4B). We then arbitrarily selected eight genes from these unannotated lncRNAs as well as two genes with annotated names that were not well studied and examined their subcellular distribution using fluorescent in situ hybridization (FISH) (Fig. 4C). Typically, we observed one or two discrete dots in the nucleus, and diffuse signals in nucleoplasm were also observed with probes that detect RP24-122I22.2 or RP23-316B4.2 (Fig. 4C). We also examined the subcellular distribution of three of the functional lncRNA candidates selected from HepG2 cells (Fig. 4A,D). To confirm the discrete dots observed for some of the lncRNAs corresponded to their transcription sites, we focused on RP11-113K21.5, which is located upstream of Rab30 and is conserved between humans and mouse (AC104921.1 in mouse) (Fig. 4E). To identify the site of transcription, we performed FISH using a probe that detects an intron of Rab30, a gene located immediately upstream of RP11-113K21.5, which should visualize the genomic locus (Fig. 4E). Transcripts of RP11-113K21.5 were observed as two discrete dots in HepG2 cells, which were closely associated with the signals detected by the Rab30 intron probe (Fig. 4E). These observations possibly implied that RP11-113K21.5 accumulated at the site of transcription and controlled the expression of neighboring genes, as described for novel lncRNAs identified in a previous study (Joung et al. 2017). To examine the half-life of these lncRNAs localizing at the putative transcription sites, we performed BRIC (bromouridine immunoprecipitation chase) analyses (Tani et al. 2012). RNAs are initially labeled with bromouridine, and the chronological decrease was examined using immunoprecipitated RNA samples with an anti-bromouridine antibody. We used HepG2 cells for this experiment because this cell type efficiently incorporated the uridine analog bromouridine, while cultured hippocampus neurons did not. Unlike the half-lives of stable transcripts, such as 18S rRNA (38.7 h) or MALAT1 (8.57 h), the half-lives of RP11-113K21.5 and RP11-46C20.1 were relatively short, 1.55 h and 1.29 h, respectively, which were comparable to the half-lives of relatively unstable NEAT1 (2.89 h) and SNHG1 (2.07 h) (Fig. 4F). These observations suggested that the functional lncRNA candidates we identified are actively transcribed but rapidly degraded in the nucleoplasm, resulting in localization at the putative transcription sites.

### LncRNAs that associate with the prion-like domain-containing RBPs exhibit larger decreases in UPA-seq reads

**FIGURE 5.**
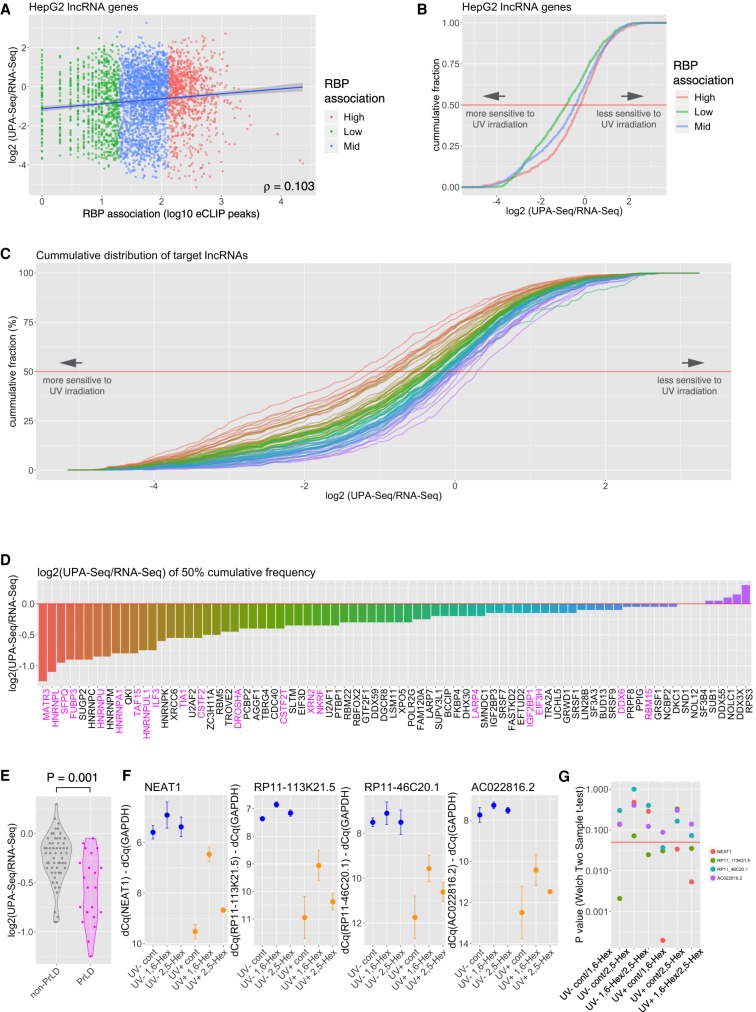
Association with PrLD-containing RBPs preferentially decreases UPA-seq reads. (*A*) Scatter plot illustrating the relationship between the total number of eCLIP peaks mapped on each transcript and the fold change of reads upon UV irradiation. Blue line represents the regression line. (*B*) Cumulative distribution of lncRNAs that possessed high (top 25% quintile, red), middle (25–75 percentile, blue), and low (bottom 25% quintile, green) total eCLIP peaks for each RBP along the fold change of reads upon UV irradiation. Note that lncRNAs with higher total eCLIP peaks tend to be represented in a group that is less sensitive to UV irradiation. (*C*) Cumulative distribution of lncRNAs bound by a series of RBPs shown in *D* along with the fold change of reads upon UV irradiation. Note that each RBP contributes differentially to the decreased UPA-seq reads upon UV irradiation. (*D*) Bar plots illustrating the fold change of reads mapped to lncRNAs at the median targeted by the indicated RBPs. The magenta color represents the name of the RBPs containing a PrLD. (*E*) Violin and quasi-random beeswarm plots showing the fold change of reads mapped to lncRNAs at the median targeted by RBPs without PrLD (non-PrLD) and with PrLD (PrLD). Bar plots indicate the mean ± SD of fold change values shown in each category. (*F*) RT-qPCR quantification of specific lncRNAs indicated at the *top* of each panel from UV-irradiated (UV+) and nonirradiated (UV−) cells pretreated with control DMSO (cont), 1,6-hexanediol (1,6-Hex), and 2,5-hexanediol (2,5-Hex). The 2,5-hexanediol treatment suppressed the reduction of recovery from the aqueous phase upon UV irradiation. The reversed *y*-axes represent the Δ Cq values of each lncRNA relative to GAPDH. Bar plots indicate the mean ± SD of three biological triplicates. (*G*) Point plots of the *P*-values shown in *F*, as calculated by Welch's *t*-test.

All of the results described above suggested that functional lncRNAs were easily crosslinked to associated proteins and that their recovery from the aqueous phase was remarkably decreased upon UV irradiation. To investigate the types of RBPs that preferentially associate with functional lncRNAs, we reanalyzed the ENCODE eCLIP (enhanced UV crosslinking and immunoprecipitation) resources (https://www.encodeproject.org/eclip/) in which the binding sites of 78 RBPs were systematically studied (Van Nostrand et al. 2016, 2017). We initially calculated the total number of eCLIP peaks mapped on each transcript and compared the values with the fold change of reads upon UV irradiation. Unexpectedly, we did not observe a correlation between the two values (Fig. 5A), suggesting that association with proteins in general did not necessarily result in decreased read counts in UPA-seq. We also examined the cumulative distribution of lncRNAs classified by RBP association (high, mid, low) along the fold change of reads (Fig. 5B). In this analysis, a group of lncRNAs with a smaller number of eCLIP peaks (low) exhibited even greater decreases in read counts in UPA-seq, suggesting that greater RBP association results in decreased crosslinking to proteins (Fig. 5B). These unexpected results led us to speculate that each RBP differentially contributed to the decrease in UPA-seq reads. We thus examined the cumulative distribution of target lncRNAs for each RBP (i.e., lncRNAs with eCLIP peaks of specific RBPs) along the fold change of reads after UV irradiation. As expected, lncRNAs targeted by each RBP were differentially represented along the fold change of reads obtained by UPA-seq (Fig. 5C,D). Interestingly, lncRNAs bound by RBPs that contain PrLDs (magenta color in Fig. 5D) exhibited a greater negative fold change in reads upon UV irradiation (Fig. 5D). Comparison of the median target lncRNAs revealed that the association of PrLD-containing RBPs significantly reduced the recovery of lncRNAs upon UV irradiation compared with the association of other RBPs (*P* = 0.037 in Wilcoxon rank sum test) (Fig. 5E).

A series of recent studies demonstrated that PrLD-containing RBPs induce liquid–liquid phase separation or hydrogel formation in vitro, providing a molecular basis for the formation of nonmembranous cellular bodies in vivo (for review, see Uversky 2017), including the paraspeckles assembled on Neat1 (for review, see Fox et al. 2018). To further test whether the phase separation of PrLD-containing proteins and associated lncRNAs facilitated covalent bond formation upon UV irradiation, we treated HepG2 cells with 1,6-hexanediol, an aliphatic alcohol that disrupts weak hydrophobic interactions and interferes with liquid–liquid phase separation (Ribbeck and Görlich 2002; Molliex et al. 2015; Strom et al. 2017). We also treated the cells with 2,5-hexanediol, which is less effective in disrupting phase-separated cellular bodies. Subsequent RT-qPCR analyses revealed that hexanediol significantly (*P* < 0.05) restored the recovery of NEAT1 as well as the novel functional lncRNA candidates RP11-113K21.5 and RP11-46C20.1 from the aqueous phase upon UV irradiation (Fig. 5F,G). These observations suggested that functional lncRNAs form tight ribonucleoprotein complexes that are easily crosslinked by UV irradiation through interactions with PrLD-containing proteins.

### Reduction of UPA-seq reads mapped to localizing mRNAs

**FIGURE 6.**
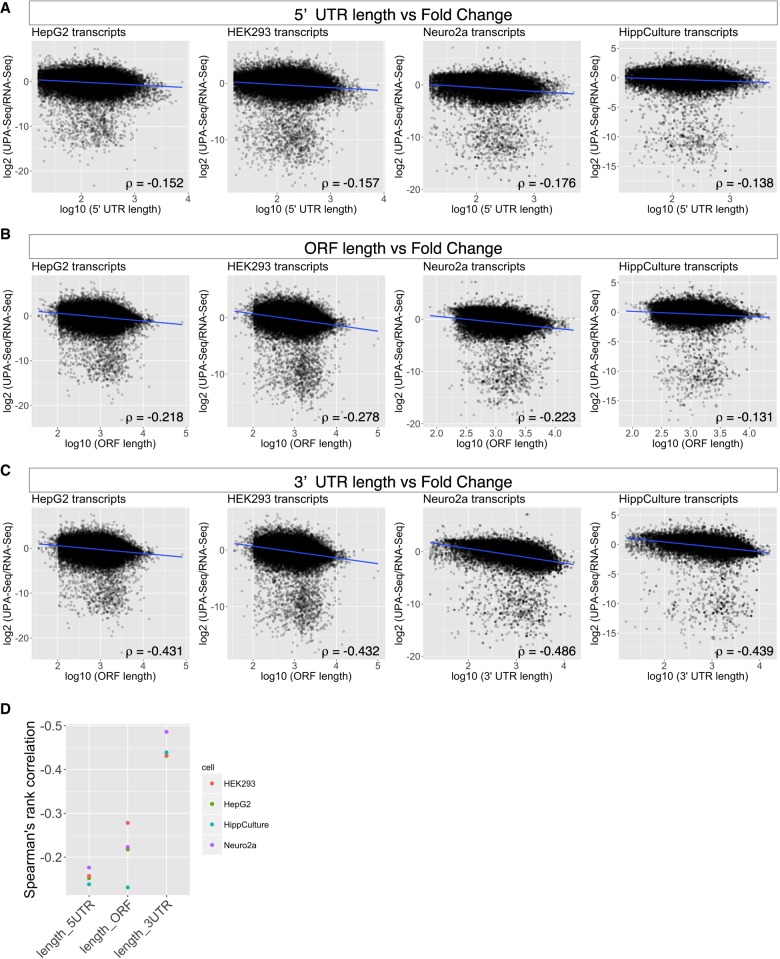
Properties of mRNAs that exhibit reduced recovery from the aqueous phase upon UV irradiation. (*A–C*) Scatter plots illustrating the relationship between the lengths of 5′ UTR (*A*), ORF (*B*), and 3′ UTR (*C*) of protein-coding mRNAs and the fold change of reads upon UV irradiation. Blue lines indicate the regression line. Correlation coefficient (ρ) values were calculated by Spearman's rank correlation analysis. (*D*) Point plots of the Spearman's rank correlation coefficient ρ value shown in *A–C*.

**FIGURE 7.**
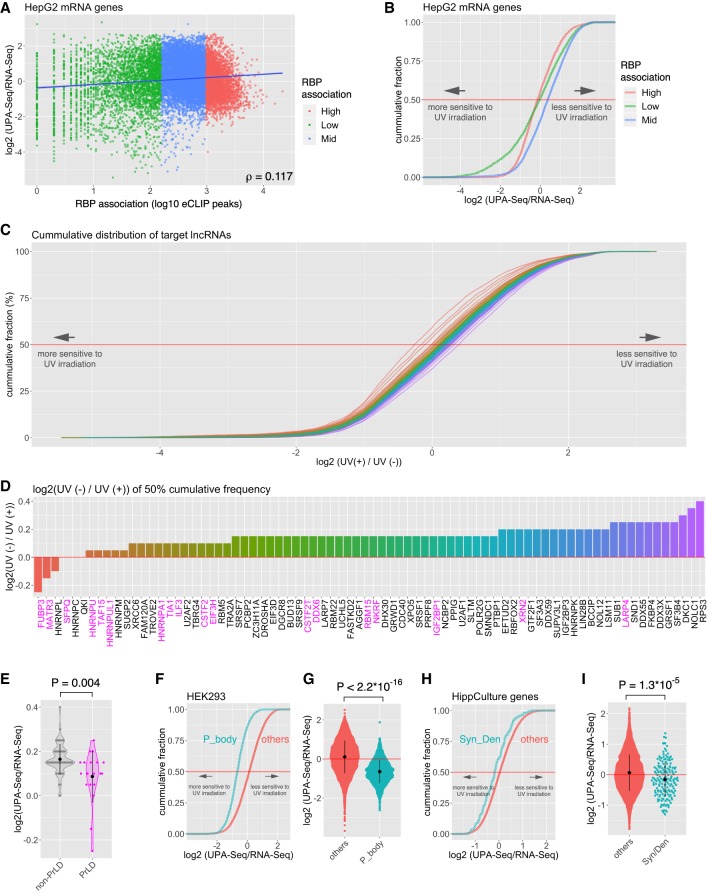
mRNAs localizing to P-bodies and synapses exhibit decreased reads in UPA-seq. (*A*) Scatter plot illustrating the relationship between the total number of eCLIP peaks mapped on each transcript and the fold change of reads upon UV irradiation. The blue line represents the regression line. (*B*) Cumulative distribution of mRNAs that possessed high (top 25% quintile, red), middle (25–75 percentile, blue), and low (bottom 25% quintile, green) total eCLIP peaks for each RBP along the fold change of reads upon UV irradiation. (*C*) Cumulative distribution of mRNAs bound by the series of RBPs shown in *D* along with the fold change of reads upon UV irradiation. (*D*) Bar plots illustrating the fold change of reads mapped to mRNAs at the median targeted by each RBP. The magenta color represents RBPs containing PrLD. (*E*) Violin and quasi-random beeswarm plots showing the fold change of reads mapped to mRNAs at the median targeted by RBPs without PrLD (non-PrLD) and with PrLD (PrLD). Bar plots indicate the mean ± SD of values in each category. (*F*) Cumulative distribution of mRNAs enriched in P-bodies (P_body) and other mRNAs (others) along with the fold change of reads upon UV irradiation. (*G*) Quasi-random beeswarm plot showing the fold change of reads mapped to mRNAs that are enriched in P-bodies (P-body) and other mRNAs (other). Note that mRNAs enriched in P-bodies exhibit a greater negative fold change compared to the others. The *P*-value was calculated by the Wilcoxon rank sum test. (*H*) Cumulative distribution of mRNAs localizing at synapse/dendrite (Syn/Den) and other mRNAs (others) along with the fold change of reads upon UV irradiation. (*I*) Quasi-random beeswarm plot showing the fold change of reads mapped to mRNAs that localize at the synapse/dendrite (Syn/Den) and other mRNAs (other). Note that synapse/dendrite-localizing mRNAs exhibit greater negative fold change compared to others. *P*-value was calculated by Wilcoxon rank sum test.

Although we observed a distinct reduction in UPA-seq reads that mapped to functional lncRNAs, a certain portion of protein-coding mRNAs also exhibited a comparable decrease in UPA-seq reads (Fig. 2A,B). To obtain insight into the physiological relevance of this reduction, we firstly compared the correlation between the fold change of reads upon UV irradiation and the length of three regions of mRNAs, the 5′ and 3′ untranslated regions (UTR) and the open reading frame (ORF) (Fig. 6A–C). While the 5′ UTR and ORF did not exhibit a strong correlation, the length of the 3′ UTR exhibited a mild (ρ > 0.4) correlation with the decrease in UPA-seq reads in all of the cell types we examined (Fig. 6A–D). As was the case for lncRNAs, we did not observe correlations between the total eCLIP peaks mapped on each mRNA and the fold change of reads in UPA-seq (Fig. 7A,B), and each RBP contributed differentially to the decrease of UPA-seq reads (Fig. 7C–E). We speculated that mRNAs were efficiently crosslinked to PrLD-containing RBPs when they are localized in phase-separated cellular bodies. To test this idea, we examined the cumulative distribution of mRNAs enriched in P-bodies, representative phase-separated nonmembranous cellular bodies (for review, see Luo et al. 2018), described in Hubstenberger et al. (2017) along with the fold change of reads upon UV irradiation in our data set. As expected, the P-body-enriched mRNAs (log_2_ fold change > 2) were preferentially represented in the group that exhibited the greatest negative fold change upon UV irradiation (Fig. 7F,G). We also examined the cumulative distribution of dendrite- or axon-localizing mRNAs described in Cajigas et al. (2012), many of which are known to localize to neuronal RNA granules (for review, see Hirokawa 2006). Again, we observed that these synaptic/dendritic neuronal mRNAs were preferentially represented in the group that exhibited a decrease in UPA-seq reads (Fig. 7H,I).

## DISCUSSION

We have thus demonstrated that comparison of UPA-seq and RNA-seq provides valuable information for the selection of candidate lncRNAs to be analyzed in subsequent studies. We recognize that the reduced recovery from the aqueous phase upon UV irradiation is not a definitive feature for “functional” noncoding RNAs because such reduction was not observed with classic functional noncoding RNAs involved in evolutionarily conserved processes of gene expression, including rRNA, UsnRNAs, snoRNAs, 7SK, and 7SL, all of which form well-characterized ribonucleoprotein complexes. Considering that covalent bond formation between the RNA and protein should impair the physiological function of these molecular machineries, nucleotide sequences and amino acid residues of these classic ribonucleoprotein complexes might have been selected to decrease the efficiency of UV crosslinking during the course of evolution. Alternatively, these classic lncRNAs are highly folded with longer double-stranded RNA structures, resulting in a decreased portion of exposed bases that interact with proteins. We also failed to detect reduction of UPA-seq reads mapped to lncRNAs, including Blustr and Upperhand, which regulate the expression of nearby genes by their transcription but not their transcribed products. Our prediction of functionality is thus applicable to a limited group of lncRNAs that tightly associate with proteins to form large ribonucleoprotein complexes. Nevertheless, we propose UPA-seq should be useful for selecting candidate genes involved in the processes of interest during the initial screening step, due to its technical simplicity and compatibility.

Although all of the cell types we used exhibited a similar reduction in UPA-seq reads, they responded differentially to UV irradiation: The fold change of reads mapped to lncRNAs in HepG2 cells and Neuro2a cells exhibited greater negative values and bimodal distributions, while HEK293 cells and HippCulture cells exhibited a milder decrease and moderate Gaussian distributions. The differential responses might represent differential adaptation of each cell line to UV irradiation through proteins specifically expressed in each cell type. Alternatively, lncRNAs receive different amounts of UV energy due to cell type-specific composition of molecules that absorb UV light, including various metabolites as well as the residues of proteins and nucleic acids that are differentially expressed. Regardless, the condition of UV irradiation must be carefully optimized for each cell type for maximal use of UPA-seq to select functional lncRNA candidates.

Importantly, lncRNAs that exhibited greater decreases in UPA-seq reads preferentially associated with RBPs containing the PrLD. A series of studies revealed that one of the characteristic functions of lncRNAs is to form nonmembranous organelles or a ribonucleoprotein milieu within the cells via liquid–liquid phase separation through interaction with PrLD-containing RBPs (Hennig et al. 2015; Banani et al. 2017; Fay et al. 2017; Fay and Anderson 2018; Maharana et al. 2018), and UPA-seq may be useful for identifying novel lncRNAs included in phase-separated cellular bodies. Actually, recently reported semi-extractable lncRNAs that form novel nuclear bodies were represented in a group that exhibited greater decreases in UPA-seq reads, and we did observe the formation of a distinct RNA localization around the putative transcription sites for RP23-316B4.2 and RP23-14P23.3. Further, functional studies on these lncRNA candidates will expand our knowledge of the biological processes regulated by this group of molecules.

While we primarily focused on lncRNAs in this study, certain fractions of mRNAs also exhibited a dramatic decrease in UPA-seq reads. Interestingly, the length of 3′ UTR exhibited good correlation with fold changes in reads upon UV irradiation, which was consistent with the well-established concept that the subcellular localization of mRNAs is regulated by 3′ UTR regions through their interaction with miscellaneous RBPs (for review, see Mayr 2016). We observed preferential representation of P-body-enriched and neurite-localizing mRNAs among the genes that exhibited greater decreases in reads upon UV irradiation, which preferentially associated with proteins containing PrLD. UPA-seq might thus be useful to make a candidate list of mRNAs that are localized in phase-separated nonmembranous cellular bodies including, stress granules, neuronal granules, and germinal granules.

Overall, the simple combination of UV irradiation and phenol-chloroform extraction provides a versatile tool for the prediction of candidate lncRNAs that tightly associate with regulatory proteins. It would be intriguing if we could predict functionality of lncRNAs in the future by combining information obtained by scalable deep sequencing analyses including UPA-seq, eCLIP, and various structure-probing technologies that have been recently developed (for review, see Silverman et al. 2016).

## MATERIALS AND METHODS

### Cell culture

HEK293T and Neuro2a cells were cultured in DMEM/Ham's F-12 (#048-29785, Wako, Japan) supplemented with 10% fetal bovine serum and penicillin-streptomycin. HepG2 cells were cultured in DMEM (#D5796, Sigma Aldrich) supplemented with 10% fetal bovine serum and penicillin-streptomycin. Primary hippocampal neurons were prepared from E16.5 C57/BL6NCr or ICR mouse brain for RNA-seq and FISH experiments, respectively, following a previously described protocol (Chiba et al. 2014). Briefly, hippocampal tissues were dissected from E16.5 embryos in ice-cold HEPES-buffered saline (HBSS), treated with 2 mg/mL papain (#LSS03119, Worthington) for 20 min at 37°C, and then gently dissociated into single cells by mild pipetting in the presence of 0.2 mg/mL DNase I (#311284932001, Roche). After filtration through a cell strainer (#352350, BD Falcon), cells were then resuspended in culture medium (Neurobasal medium, #21103049, Thermo Fisher) supplemented with B27 (#17504044, Thermo Fisher), L-glutamine, and 1% horse serum and then plated over coverslips precoated with poly-L-lysine and polyethylenimine at a density of 3.5 × 10^4^ cells/cm^2^. Three days after the plating, cells were treated with 10 µM AraC for 24 h to remove the glial cells and then further cultured for 10 d. For hexanediol treatment, HepG2 cells were treated with 15% 1,6- or 2,5-hexanediol (#240117 and H11904, SIGMA) or DMSO for 5 min at room temperature. After treatment, the medium was replaced with HCMF, and the cells were immediately irradiated with UV as described below.

### UV irradiation and RNA extraction

Culture medium was replaced with 1 mL of ice-cold HBSS, and cells were crosslinked at 254 nm UV with 120 mJ/cm^2^ total energy in a Funa UV linker (#FS-800, Funakoshi, Japan) on ice. Total RNA was purified using TRIzol reagent (#15596026, Invitrogen) following the manufacturer's instructions with an additional heating step at 55°C for 10 min before the addition of 1/5 volume of chloroform.

### RT-qPCR analyses

cDNA was synthesized from 1 µg of total RNA using the ReverTra Ace qPCR RT Master Mix (#FSQ-201, TOYOBO, Japan) following the manufacturer's instruction. A total of 1/50 of synthesized cDNA and 0.3 µM for each primer were used for the qPCR reactions. RT-qPCR was performed using THUNDERBIRD SYBR qPCR Mix (#QPS-201, TOYOBO) and CFX Connect (BIORAD) with the following conditions: 95°C for 1 min followed by 40 cycles of 95°C for 15 sec and 60°C for 60 sec. The qPCR primers used in this study are listed in Supplemental Table S1.

### Half-life analyses of candidate functional lncRNAs with BRIC

BRIC was performed using a BRIC kit (#RN1007, MBL) according to the manufacturer's recommendations. Briefly, cells were incubated in medium containing 150 µM 5-bromo-uridine (BrU) for 24 h. After replacing the BrU-containing medium with a BrU-free medium, cells were harvested at each time point (0, 2, and 8 h). TRIzol reagent (#15596026, Invitrogen) was used to extract total RNA from each sample, and target-gene expression levels were quantified by RT-qPCR. Normalization was performed using Spike-in control.

### RNA extraction with proteinase K pretreatment

After UV irradiation, HBSS was replaced with 200 µL proteinase K solution (1% SDS, 10 mM Tris-HCl [pH 8.0], 10 mM EDTA [pH 8.0] + 1 mM DTT + 20 µg/mL proteinase K [#3115887001, Roche]), and the suspended samples were collected into 1.5 mL tubes and further incubated at 37°C for 30 min. TRIzol LS reagent (#10296010, Invitrogen) was used to extract the total RNA from each sample.

### Northern blot analyses

10 µg of total RNAs were separated on 1% agarose gel containing 3% formaldehyde, rinsed in distilled water, denatured in 0.02 N NaOH for 20 min, equilibrated in 20× SSC, and blotted on a nylon membrane following standard procedure. DIG-labeled probes were hybridized in DIG Easy Hyb (Roche, #11603558001) at a concentration of 0.1 µg/mL, and hybridized probes were detected with alkaline phosphatase-conjugated anti-DIG antibodies (Roche, #11093274910) and CDP-star (Roche, #CDP-RO). Chemiluminescence signals were detected with the ChemiDoc Touch Imaging System (Bio-Rad) and quantified using the ImageJ software.

### FISH

FISH was performed following a previously described protocol (Mito et al. 2016). Briefly, cells were plated on PLL-coated coverslips and fixed in 4% paraformaldehyde for 10 min at room temperature. After permeabilization with 0.5% Triton X-100 in PBS, cells were hybridized with 1 µg/mL DIG- or FITC-labeled RNA probes diluted in hybridization buffer overnight at 55°C. After washing twice with 50% formamide/2× SSC for 30 min at 55°C, samples were treated with RNaseA, and further washed with 2× SSC and 0.2× SSC for 30 min each at 55°C. Fluorescent images were obtained using an epifluorescence microscope (BX51, Olympus) equipped with a CCD camera (DP70). The probes and antibodies used are described in Supplemental Table S1.

### RNA-seq and data analyses

Deep sequencing libraries were made following a standard protocol using a Ribo-Zero Gold rRNA Removal Kit (Human/Mouse/Rat) (Illumina) and TruSeq RNA Sample Preparation v2 (Illumina). Deep sequencing was performed using an Illumina Hiseq2500 (Neuro2a, HippCulture, and 293 cells) or Hiseq4000 (HepG2 cells). Sequence reads were mapped to hg38 and mm10 genome builds using TopHat2 (Kim et al. 2013). The numbers of sequence reads mapped to each exon were counted using featureCount (Liao et al. 2014) with -O option (allowMultiOverlap) and analyzed at the gene level. After removing genes that have an average read of 0.5 per millions of reads in UV nonirradiated samples, the data count was analyzed using DESeq2 (Love et al. 2014) to calculate the normalized fold change between the UV-irradiated and nonirradiated samples. For the numbers of reads mapped to each transcript, we used Cuffdiff2 (Trapnell et al. 2013), and low expressers (RPKM < 0.1) were removed for subsequent correlation analyses. The annotations of genes and transcripts were obtained from the GENCODE homepage (https://www.gencodegenes.org/), and GRCh38.p12 and GRCm38.p6 were used for the human and mouse genome annotation, respectively. The lengths of lncRNAs were calculated from the Fasta files downloaded from the GENCODE homepage (lncRNA transcript sequences) using the readDNAstringSet command in the Biostrings package (DOI: 10.18129/B9.bioc.Biostrings). The length of the 5′ UTR, 3′ UTR, and ORF of mRNAs was obtained from the foldUtr5, knownGenePep, and foldUTR3 tables in the GENCODE/UCSC genes track for human/mouse, which was downloaded using the Table Browser of the UCSC genome browser (http://genome.ucsc.edu/index.html). Coverage plots, beeswarm plots, scatter plots, bar plots, cumulative plots, and violin plots were drawn with ggplot2 (Wickham 2009). For the eCLIP analyses, bed files for each RBP (narrow peaks) were downloaded from the ENCODE homepage (https://www.encodeproject.org/search/?type=Experiment&assay_title=eCLIP), converted to bam files using bedtools (Quinlan and Hall 2010), and the number of peaks for each transcript was counted using featureCounts. As for the cellular fraction data of HepG2 cells, the count data of the nuclear (ENCSR061SFU) and cytoplasmic (ENCSR862HPO) rRNA-depleted RNA-seq were downloaded from the ENCODE homepage.

## DATA DEPOSITION

The sequencing data obtained in this study were deposited at NCBI GEO (GSE114789). To review GEO accession GSE114789, go to https://www.ncbi.nlm.nih.gov/geo/query/acc.cgi?acc=GSE114789.

## SUPPLEMENTAL MATERIAL

Supplemental material is available for this article.

## Supplementary Material

Supplemental Material
