# Supplementary material for: UPA-seq: prediction of functional lncRNAs using differential sensitivity to UV crosslinking
Source: RNA. 2018 Dec;24(12):1785–802. doi: 10.1261/rna.067611.118 (PMC6239193; doi:10.1261/rna.067611.118)
Supplement: Supplemental Material [file supp_24_12_1785__index.html]

UPA-Seq: Prediction of Functional LncRNAs Using Differential Sensitivity to UV Crosslinking — UPA-seq: prediction of functional lncRNAs using differential sensitivity to UV crosslinking — Supplemental Material 

# UPA-seq: prediction of functional lncRNAs using differential sensitivity to UV crosslinking

## Supplemental Material

- Supplemental\_Table\_T1.xlsx
